# Enteral nutrition protects children undergoing allogeneic hematopoietic stem cell transplantation from blood stream infections

**DOI:** 10.1186/s12937-020-00537-9

**Published:** 2020-04-10

**Authors:** Daniele Zama, Edoardo Muratore, Elena Biagi, Maria Luisa Forchielli, Roberto Rondelli, Marco Candela, Arcangelo Prete, Andrea Pession, Riccardo Masetti

**Affiliations:** 1 Paediatric Oncology and Haematology Unit ‘Lalla Seràgnoli’, Department of Paediatrics, University of Bologna, Sant’Orsola Malpighi Hospital, Via Massarenti, 11, 40138 Bologna, Italy; 2 Department of Pharmacy and Biotechnology, University of Bologna, Bologna, Italy; 3 Department of Paediatrics, Medical School, Bologna, Italy

**Keywords:** Enteral nutrition, Parenteral nutrition, Hematopoietic stem cell transplantation, Paediatrics, Blood stream infections, Gut microbiota

## Abstract

Enteral Nutrition (EN) is recommended as first line nutritional support for patients undergoing Allogeneic Hematopoietic Stem Cell Transplantation (allo-HSCT), but only few studies exist in the literature which compare EN to Parenteral Nutrition (PN) in the paediatric population.

Forty-two consecutive paediatric patients undergoing allo-HSCT at our referral centre between January 2016 and July 2019 were evaluated. Post-transplant and nutritional outcomes of patients receiving EN for more than 7 days (EN group, *n* = 14) were compared with those of patients receiving EN for fewer than 7 days or receiving only PN (PN group, *n* = 28). In the EN group, a reduced incidence of Blood Stream Infections (BSI) was observed (*p* = 0.02) (*n* = 2 vs. *n* = 15; 14.3% vs. 53.6%). The type of nutritional support was also the only variable independently associated with BSI in the multivariate analysis (*p* = 0.03). Platelet engraftment was shorter in the PN group than in the EN group for a threshold of > 20*10^9^/L (*p* = 0.04) (23.1 vs 35.7 days), but this correlation was not confirmed with a threshold of > 50*10^9^/L. The Body Mass Index (BMI) and the BMI Z-score were no different in the two groups from admission to discharge.

Our results highlight that EN is a feasible and nutritionally adequate method of nutritional support for children undergoing allo-HSCT in line with the present literature. Future functional studies are needed to better address the hypothesis that greater intestinal eubyosis maintained with EN may explain the observed reduction in BSI.

## Introduction

Allogeneic Hematopoietic Stem Cell Transplantation (allo-HSCT) is a potentially curative strategy for many oncological, haematological, metabolic and immunological diseases in children. It includes a conditioning regimen with chemotherapy, radiotherapy and immunotherapy, allowing patients to receive donor haematopoietic stem cells [1, 2]. Despite the recent advances in the field, the procedure is still associated with marked morbidity and mortality [3], mainly due to recurrence of the primary disease or to transplant-related complications. The two leading complications are infections, particularly bacterial blood stream infections (BSI), and acute Graft versus Host Disease (aGvHD) [2, 3]. The latter is characterised by the response of alloreactive donor T cells to host organs including the skin, gut and liver [4] while infections are favoured by prolonged immunosuppression and mucosal damage [2].

The majority of children start treatment in a relatively healthy nutritional status [5]. Oral feeding in the early post-transplant period is impaired because of conditioning regimen-side effects, mainly vomiting, anorexia, diarrhoea and mucositis. The reduction in caloric intake gives rise to a rapid deterioration of the nutritional status [5–7] which is associated with lower overall survival as well as higher complication rates during treatment [8–10].

Historically, parenteral nutrition (PN) was considered to be the method of choice for the nutritional support of patients undergoing allo-HSCT [11]. However, in light of PN-related complications (sepsis, metabolic and hepatic disorders, gut mucosal atrophy) [12], some concerns have been raised regarding its use [13–16]. Another feeding option is Enteral Nutrition (EN) which involves the administration of nutrients directly into the digestive system via a nasogastric tube (NGT), and has been shown to be feasible and effective in other clinical settings [17–19]. Currently, the international guidelines provided by European Bone Marrow Transplantation and ESPEN recommend EN as first line nutritional support for patients undergoing allo-HSCT [20, 21]. Nevertheless, a recent survey has shown that the majority of European transplant centres still use PN over EN, mainly due to the constant availability of central venous access [22]. Another possible explanation is the preference given to PN by caregivers because of the perceived invasiveness of EN [23].

In the paediatric population, the few studies comparing EN to PN involving a limited number of patients, found that EN had potential benefits regarding aGvHD and platelet engrafment [24]. Considering the increasing knowledge concerning the role of gut microbiota dysbiosis in the development of main complications after HSCT, these benefits may be potentially explained by the maintenance of gut eubyosis and epithelium integrity in patients receiving EN [25].

Thus, the aim of this study was to evaluate the role of EN regarding clinical and nutritional outcomes in paediatric allo-HSCT recipients and to comprehend the impact of the type of nutritional support on the main complications of allo-HSCT.

## Methods

### Patients

Consecutive patients undergoing allo-HSCT for either malignant or non-malignant disease were evaluated at the Paediatric HSCT Unit of Sant’Orsola Hospital in Bologna, Italy, between January 2016 and July 2019. The study protocol was approved by the University of Bologna Ethics Committee (ref. number 19/2013/U/Tess). Written informed consent authorising the medical records to be used for research purposes was obtained from each enrolled patient or parent/legal guardian before data collection in accordance with the Declaration of Helsinki.

### Transplantation procedure

Conditioning regimen and aGvHD prophylaxis were adopted according to national and international guidelines, based on patient age, comorbidities, disease, donor and stem cell source. Conditioning regimens were divided into Myeloablative conditioning (MAC) or non-Myeloablative conditioning (no-MAC) based on the intensity level of the myeloablation provided [3]. Granulocyte-Colony Stimulating Factor (G-CSF) was administered according to the patient’s disease and haematological status.

The patients were treated in high efficiency particulate air filtered rooms in order to prevent infections. Antibiotic prophylaxis (ABP) or gut decontamination were not used for any patient.

The presence or absence of cytomegalovirus and Epstein-Barr virus were checked in both donor and recipient.

In the case of febrile neutropenia, patients were treated using empiric broad-spectrum intravenous antibiotics, with ceftazidime as the first choice, after obtaining blood cultures from a peripheral and a central line.

### Nutritional support during the neutropenic period

In allo-HSCT performed before 2018, only PN was utilised while, starting from January 2018, EN was proposed as the first choice of nutritional support during the neutropenic period. Parenteral nutrition was started as soon as the patient could not eat properly by mouth with a hospital-made compound administered by means of a Central Venous Catheter (CVC). The compound used did not have a standardised composition, but the hospital pharmacy regularly provided a customised formula based on the prescription of the physician for each patient. Nutritional needs were calculated based on the weight of the children. The parenteral energy goal was reached progressively within 2–4 days. The concentration of glucose given varied from 5.0% to 12.5%. Calories derived from glucose represented 70% of the non-proteic calories. The remaining 30% were derived from lipids given in the form of soybean oil emulsion. Proteins were given at a dose of 1 g/kg/day. Oligoelements, vitamins and electrolytes were also administered as supplements.

The EN was administered via a nasogastric tube (NGT) using a commercial formula. Information regarding the enteral mixture administered is reported in Supplementary Table S1. The families received appropriate information regarding EN and NGT positioning during a pre-transplantation interview. Depending on the child’s age, the patient also received information. The NGT was inserted between days − 2 and + 2 from HSCT. The caloric goal was based on the basal metabolism rate, calculated according to the Schofield formula [26, 27]. To improve tolerability, the starting dose was lower than the calculated goal, and scaled up in the following days depending on patient tolerance. The EN was administered either continuously, with a break during the night, or by bolus.

Patients who did not tolerate EN due to vomiting, nausea, diarrhoea, abdominal discomfort, gastric residuals or NGT intolerance were switched to PN. In the case that an inadequate amount of calories were delivered with EN, supplementation with an intravenous glucose solution or PN was administered. The EN and PN were stopped as soon as the patient was able to eat on his/her own.

### Study design, transplantation and nutritional outcomes

**Fig. 1:**
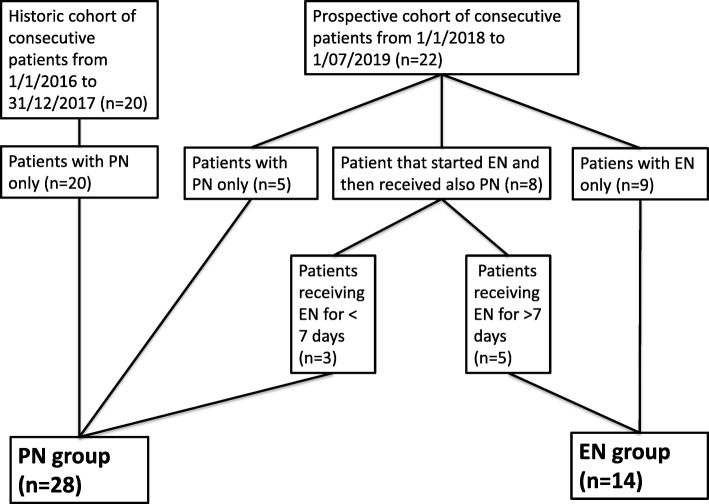
Flowchart of subject recruitment and nutritional support modalities. *Abbreviations: EN = Enteral Nutrition; n = number of patients; PN = Parenteral Nutrition*

Children receiving EN for more than 7 days were included in the EN group while children receiving EN for fewer than 7 days or receiving only PN were included in the PN group (Fig. 1). Patients who received a second allo-HSCT and/or those having previous chronic bowel disease were excluded from this study.

In order to assess the effect of the route of nutritional support on clinical and nutritional outcomes, certain data were recorded from admission to discharge. Neutrophil and platelet engraftments were defined as 3 consecutive days with a count > 0.5 *10^9^/L and 7 consecutive days transfusion free with a count > 20*10^9^/L or > 50*10^9^/L, respectively. Oral mucositis occurrence was evaluated by a nurse and a physician, and graded according to the World Health Organization (WHO) criteria [28]. Acute graft versus host disease was diagnosed on the basis of clinical symptoms and graded from 0 to IV according to the Glucksberg classification [29]. Steroid resistant gut aGvHD was defined as progression within 3–5 days of starting corticosteroid treatment or an incomplete response by 7–14 days [21].

Veno-occlusive disease was diagnosed according to the recent paediatric criteria proposed by the European Society for Blood and Marrow Transplantation [30]. Blood stream infections (BSI) were considered only when confirmed by a positive bacterial blood culture. The duration of fever and antibiotic therapy was evaluated only in the first 30 days, based on the theory that, in this time span, infections were mainly caused by the patient’s endogenous bacterial flora [21]. Transplant-related mortality was assessed on day 100 after transplantation.

Body weight was recorded at admission, once a week from the day of the HSCT to day + 35 and at discharge in order to evaluate the effect of the nutritional support. Maximum weight loss was calculated by subtracting the lowest weight recorded during hospitalisation from the baseline value (considered that of the day of HSCT). Weight loss > 10% of the baseline value was then considered for the analysis.

The Body Mass Index (BMI) was then calculated using the following formula: BMI=Weight (kg)/ Height^2^ (cm). The height considered for the calculation was that recorded at admission. The BMI Z-score was calculated for children from 2 to 20 years of age using the online calculator of “The Children Hospital of Philadelphia” [31], based on Centers for Disease Control and Prevention growth charts [32]. For patients under 2 years of age, the Z-score was obtained using WHO growth charts [33]. Albumin supplements, hypophosphatemia (< 2.5 mg/dl) and the elevation of *gamma-glutamyltransferase* (> 24 U/L) and direct bilirubin (> 0.30 mg/dl) were reported in order to investigate potential adverse effects of PN.

### Statistical analysis

Qualitative variables were compared using Fisher’s exact test while means were compared using the t-test corrected in case of unequal variances. Only *p* values < 0.05 were considered to be statistically significant. Transplant-related mortality (TRM) Kaplan-Meier curves were compared with log-rank test while cumulative incidence of BSI and aGvHD was calculated using the Kalbfleisch and Prentice method and was compared using the Gray test.

In order to investigate the variables associated with the incidence of BSI, univariate and multivariate analyses were carried out using logistic regression, entering only variables considered statistically significant (*p* < 0.05) in the univariate analysis into the multivariate analysis.

## Results

Forty-two patients were enrolled, 14 and 28 in the EN and PN groups, respectively. Enteral nutrition was proposed for 22 patients of whom 64% (*n* = 14/22) received it for more then 7 days.

The two groups were homogeneous as no statistically significant differences were found regarding age, gender, diagnosis (malignant/non-malignant), disease status at transplant (remission/no remission), donor (Matched Familial, Matched Unrelated, Mismatched Unrelated, haploidentical), infused stem cell dose, source of stem cells (bone marrow, peripheral blood, cord blood), sex-matching between the donor and the recipient, conditioning regimen (MAC/no MAC), GvHD prophylaxis. The BMI and the Z score before transplantation were also comparable in the two groups (Table 1).

**Table 1:** Patient characteristics at admission and transplantation modalities by group

	**EN group (*n*** **= 14)**	**PN group (*n*** **= 28)**	**p**
Age: months, mean (range)	131.2 (4.1–251.4)	132.4 (10.3–213.1)	NS
Sex: M/F, n,	4/10	16/12	NS
Diagnosis of Malignant Disease, n,(%)	12 (85.7)	23 (82.1)	NS
CR at transplant for malignant disease,n, (%)	8 (61.5)	17 (70.8)	NS
Donor, n, (%):			NS
• Sibling HLA identical	1 (7.2)	5 (17.9)	
• MUD	7 (50)	19 (67.8)	
• MMUD	3 (21.4)	1 (3.6)	
• Haploidentical	3 (21.4)	3 (10.7)	
Stem cell source, n, (%):			NS
• BM	12 (85.8)	26 (92.9)	
• PBSC	1 (7.1)	2 (7.1)	
• CB	1 (7.1)	0 (0)	
Sex Mismatch, n, (%)	7 (50)	15 (53.6)	NS
Infused stem cell dose, median (x10^8^ MNC/kg)	4.71	4.66	NS
Conditioning regimens, n, (%):			
• MAC	13 (92.9)	23 (82.1)	NS
• With ATG	10 (71.4)	20 (71.4)	NS
BMI, mean	17.56	18.76	NS
BMI Z-score, mean	−0.37	−0.06	NS

Abbreviations: *EN=* Enteral Nutrition, *PN=* Parenteral Nutrition *NS=* Not statistically Significant, *CR=* Complete Remission, *HLA=* Human Leucocyte Antigen, *MUD=* Matched Unrelated Donor, *MMUD=* Mismatched Unrelated Donor, *BM* = Bone Marrow, *PBSC=* Peripheral Blood Stem Cell, *CB=* Cord Blood, *MNC=* Mononuclear Cells, *MAC=* Myeloablative Condioting, *ATG=* Anti-Thymocite Globulin, *BMI=* Body Mass Index

*Statistical analysis used to calculate the p value: qualitative variables were compared using Fisher’s exact test, while means were compared with t-test corrected in case of unequal variances. p < 0.05 were considered statistically significant*

**Fig. 2:**
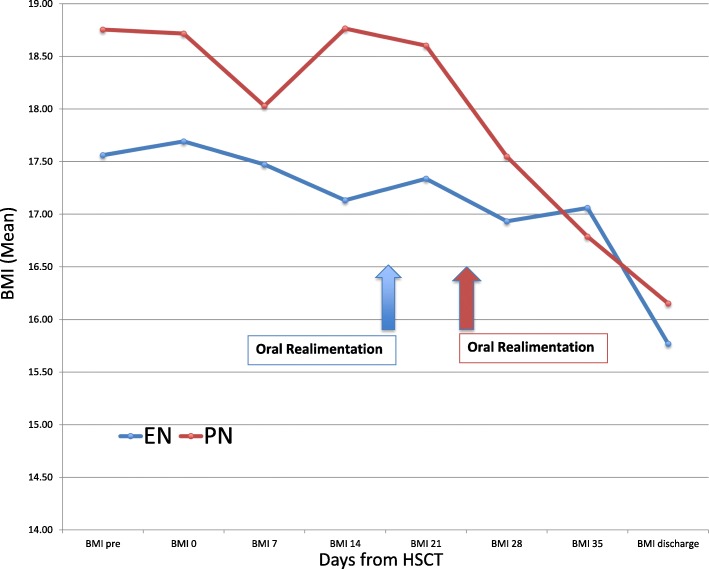
Mean BMI during hospitalisation in the EN and PN groups. There was no statistically significant difference in BMI and BMI Z-score at any time point between the EN group and the PN group from admission to discharge. The timing of start of the oral realimentation is highlighted in the figure. *Abbreviations: BMI = Body Mass Index; EN = Enteral Nutrition; HSCT = Haematopoietic Stem Cell Transplantation; PN = Parenteral Nutrition*

Although no statistically significant difference was found regarding nutritional outcomes, some noteworthy dissimilarities were observed in the two groups. The duration of the nutritional support was shorter in the EN group (*p* = 0.09) (Median: 20.7 vs. 30.7 days; Range: 12–28 vs. 10–156). Earlier oral realimentation was also observed in the EN group (*p* = 0.08) (20.9 vs. 27 days) (Table 2). The BMI and the BMI Z-score during hospitalisation were not statistically significantly different between the two groups, but there was a greater reduction in the BMI and the BMI Z-score in the PN group after oral realimentation (Fig. 2).

**Table 2:** Post-transplant outcome relative to nutritional support

	**EN group (*n*** **= 14)**	**PN group (*n*** **= 28)**	**p**
**Engrafmen**t:			
Neutrophil, days, mean	17.31	16.86	0.78
PLT > 20*10^9^, days, mean	35.67	23.14	0.04
PLT > 50*10^9^, days, mean	36.40	30,89	0.45
Duration of G-CSF, days, mean	11.62	10.46	0.51
**Oral mucositis**:			
Grade 3–4, n, (%)	5 (35.7)	3 (10.7)	0.10
Duration, days, mean	13.46	13.54	0.98
**aGvHD:**			
Grade I-IV, n, (%)	6 (42.9)	13 (46.4)	1.00
Grade III-IV, n, (%)	1 (16.7)	5 (38.46)	0.52
Gut aGvHD, n, (%)	4 (28.6)	8 (28.6)	1.00
Gut aGvHD +++/++++, n, (%)	0 (0)	5 (17.9)	0.08
Steroid Resistant Gut aGvHD, n, (%)	0 (0)	6 (21.4)	0.06
**Infections:**			
BSI, n, (%)	2 (14.3)	15 (53.6)	0.02
Duration of fever in the first 30 days post HSCT, days, mean	8.00	11.21	0.07
Length of Antibiotic therapy in the first 30 days post HSCT, days, mean	18.54	19.38	0.65
**VOD**, n, (%)	2 (14.3)	5 (17.9)	1.00
**Length of hospital stay**, days, mean	71.8	60.1	0.28
**Transfer to ICU**, n, (%)	2 (14.3)	3 (10.7)	1.00
**Nutritional Support:**			
Length of nutritional support, days, mean	20.71	30.71	0.09
Start of oral realimentation, days, mean	20.85	26.96	0.08
**Nutritional Parameters**			
Lowest weight, Kg, mean	29.78	36.63	0.31
Maximum weight loss %, mean	11.5	9.9	0.44
Weight loss > 10%, n, (%)	7 (50)	9 (32.2)	0.30
**Metabolic Parameters:**			
Albumin transfusions, U, mean	6,31	13.79	0.14
Hypophosphatemia, n, (%)	8 (57,1)	21 (75)	0.10
γGT and/or direct bilirubin elevation, n, (%)	11 (78,6)	24 (85.7)	0.26

Abbreviations: *EN=* Enteral Nutrition, *PN=* Parenteral Nutrition, *PLT=* Platelets, *G-CSF=* Granulocyte-Colony Stimulating Factor, *aGvHD=* Acute Graft versus Host Disease, *BSI=* Blood Stream Infections, *HSCT=* Hematopoietic Stem Cell Transplantation, *VOD=* Veno Occlusive Disease, *ICU=* Intensive Care Unit, *γGT=* Gamma.glutamyltransferase

*Statistical analysis used to calculate the P value: qualitative variables were compared using Fisher’s exact test, while means were compared with t-test corrected in case of unequal variances. p < 0.05 were considered statistically significant and underlined in the table*

**Fig. 3:**
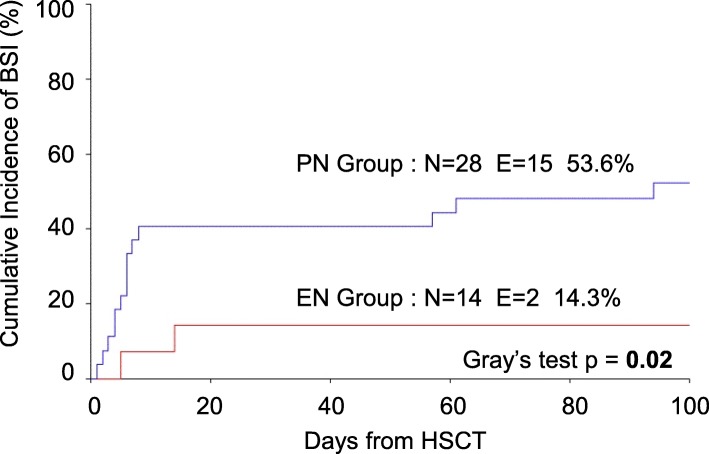
Cumulative Incidence of BSI in the EN and PN groups. Cumulative incidence was calculated using the Kalbfleisch and Prentice method and compared using the Gray test. *Abbreviations: BSI = Blood Stream Infection; E = number of Events; EN = Enteral Nutrition; HSCT = Haematopoietic Stem Cell Transplantation; N = Number of patients; PN = Parenteral Nutrition*

In the EN group, a reduced incidence of BSI was observed (*p* = 0.02) (*n* = 2 vs. *n* = 15; 14.3% vs. 53.6%). This was also confirmed by the analysis of cumulative incidence of BSI (p = 0.02) (Fig. 3). The following variables were tested in the univariate analysis to better investigate their association with the incidence of BSI: age, diagnosis (malignant/non-malignant), disease status at transplant (remission/no remission), donor (Matched Familial, Matched Unrelated, Mismatched Unrelated, haploidentical), infused stem cell dose, source of stem cells (bone marrow, peripheral blood, cord blood), sex-matching, conditioning regimen (MAC/no MAC), aGvHD prophylaxis, nutritional group (EN group vs. PN group), duration of nutritional support, maximum weight loss, number of albumin transfusions, neutrophil engraftment, days of G-CSF administration, incidence and grading of aGvHD, incidence and duration of mucositis and length of hospital stay.

The incidence of BSI was only significantly affected by the type of conditioning regimen (MAC/no MAC) (*p* = 0.04) and the nutritional group (EN group vs. PN group) (*p* = 0.02). Notably, the multivariate regression model confirmed the type of nutritional support (EN group vs. PN group) (*p* = 0.03) as the only variable independently associated with BSI.

A shorter duration of fever in the first 30 days post-transplant was also observed in the EN group, even though not statistically significant. (*p* = 0.07) (8 vs.11.2 days). No difference was found in the length of antibiotic therapy during the same time span (*p* = 0.65) (18.5 vs. 19.4). (Table 2).

Time to neutrophil engraftment and days of G-CSF administration were not different between the groups. A longer time to platelet recovery > 20*10^9^/L was observed in the EN group (*p* = 0.04) (35.7 vs. 23.1 days) but the correlation was not confirmed considering time to platelet recovery > 50*10^9^/L.

No difference was found regarding the occurrence of aGvHD between the two groups, although a trend in the EN group was observed for reduced incidence of severe gut aGvHD (Grade III-IV) (*p* = 0.08) and steroid-resistant gut aGvHD (*p* = 0.06).

No difference was found in terms of grade 3–4 and duration of mucositis, incidence of veno-occlusive disease, transfer to the intensive care unit, length of hospital stay and TRM.

## Discussion

The present study showed that EN was a feasible and nutritionally adequate method of nutritional support, with adherence to treatment comparable to other paediatric experiences reported in the literature [13, 34, 35]. No statistically significant difference was observed between the two groups regarding BMI, BMI Z-score and weight loss during hospitalisation, supporting the idea that EN may at least be comparable to PN in terms of nutritional support. Currently, there is no consensus regarding which parameter best determines malnutrition in children undergoing allo-HSCT. Body weight and BMI are easily applicable in clinical practice, but lack information on body composition and are heavily influenced by hydro-electrolytic imbalance occurring during the procedure, thus not directly reflecting the patient’s nutritional status [5, 6, 36, 37]. Additional studies comparing laboratory parameters, such as retinol-binding protein, or more detailed anthropometric measures, for example triceps skin fold thickness, are needed to better understand the influence of the type of nutritional support on overall patient nutritional status.

This paper is the first to cite evidence regarding the paediatric population that EN may protect from BSI in allo-HSCT patients not receiving ABP. The rate of BSI in the PN group was superimposable to that reported in other cohorts [13, 38]. The Authors speculated that the reduction in BSI not observed in other experiences could be due to the use of ABP potentially overlapping the protective role of EN.

Central venous catheter-related infections and the translocation of bacteria from the endogenous intestinal flora to the bloodstream represent the two main sources of bacterial infections in HSCT recipients [21]. Central venous catheter-related infectious risk may be reduced in the EN group due to the reduced use of PN, with a lower frequency in CVC handling [12]. Furthermore, PN may induce gut mucosal atrophy and greater dysbiosis [25, 39], thus promoting bacterial translocation through the intestinal mucosal barrier, and increasing the risk of intestinal bacterial domination by pathogens connected to BSI [40]. The protective role of EN from BSI could be associated with the trophic effect on the gut epithelium, either directly due to a greater presence of nutrients in loco*,* or indirectly via the production of short chain fatty acids from the gut microbiota [41, 42]. Another possible mechanism involved in the reduction of bacterial translocation is the maintenance of a thicker mucin layer in enterally fed patients [43].

The other main experiences comparing EN to PN reported in the literature showed a reduced incidence of aGvHD and, in particular, gut aGVHD [13, 38] which may also be linked to the potential reduction in dysbiosis in patients receiving EN. The data in the present study regarding gut aGvHD was in line with the results mentioned above, but failed to reach statistical significance. A larger sample size may be needed to point out the possible effect of EN on aGvHD .

The hypothesis that EN maintained a greater intestinal eubyosis after allo-HSCT has been confirmed by two recent studies. The first by Andersen et al. involved adult patients receiving ABP; it found no difference in microbial diversity, but a greater abundance of taxa associated with the increased production of short chain fatty acids, such as *Faecalibacterium*, in patients who predominantly received EN [44]. D’Amico et al. analysed longitudinally the trajectory of the compositional and functional recovery of gut microbiota in twenty paediatric patients undergoing HSCT from the same cohort in this study, of which ten were fed post-transplant with EN and ten with total PN. They observed the prompt recovery of a structural and functional eubiotic gut microbiota and short chain fatty acid layout after the disruption induced by the allo-HSCT procedure only in EN patients [45]. These results seemed to support the hypothesis that the reduced incidence of BSI observed in this study may also be explained by the eubiotic effect that EN has on the gut microbiome, promoting homeostasis of the intestinal ecosystem.

In this regard, it has not been established what role the duration of nutritional support with EN played on protecting the endogenous flora. To date, no study exists in the literature evaluating how long EN should be administered with the aim of inducing a protective effect on the microbiome. Therefore, the Authors arbitrarily decided to consider a duration of EN lasting for good part of the neutropenic phase after transplantation. However, additional studies are needed to address the significant variables connected to EN, namely duration, composition of the formula and caloric content.

## Conclusions

The results in the present study showed a potentially beneficial role of EN as the first choice of nutritional support in children undergoing allo-HSCT, with a reduction in the incidence of BSI. Given the perceived invasiveness of the method and its challenges, EN should be implemented by a motivated and committed multidisciplinary team, composed of paediatric haematologists, gastroenterologists, nurses and dieticians.

Additional studies involving a larger number of patients are needed in the specific paediatric allo-HSCT setting to confirm the potential benefits of EN over PN. In this regard, it is hoped that a multicentre randomised controlled study could be arranged in the near future involving a paediatric population, mirroring the one currently ongoing in adult patients [46], in order to give further evidence supporting the use of EN in paediatric allo-HSCT recipients. Additional functional gut microbiota analyses are also needed to better address the hypothesis that a maintained greater intestinal eubyosis may explain the observed reduction in BSI, or aGvHD, in transplanted children receiving EN.

## Supplementary information

**Additional file 1.** Composition of the enteral nutrition formula.

**Additional file 2.** Mean BMI Z-score during hospitalization by group.

## Data Availability

The datasets used and/or analysed during the current study are available from the corresponding author on request.

## Abbreviations

ABP: Antibiotic prophylaxis
aGvHD: Acute graft versus host disease
BMI: Body mass index
BSI: Blood stream infections
CVC: Central venous catheter
EN: Enteral nutrition
G-CSF: Granulocyte-colony stimulating factor
HSCT: Hematopoietic stem cell transplantation
MAC: Myeloablative conditioning
NGT: Nasogastric tube
PN: Parenteral nutrition
TRM: Transplant related mortatlity
WHO: World health organization
