# Supplementary material for: Enteral nutrition protects children undergoing allogeneic hematopoietic stem cell transplantation from blood stream infections
Source: Nutr J. 2020 Apr 10;19:29. doi: 10.1186/s12937-020-00537-9 (PMC7149876; doi:10.1186/s12937-020-00537-9)
Supplement: Supplementary file 1 — Additional file 1. Composition of the enteral nutrition formula. [file 12937_2020_537_MOESM1_ESM.pptx]

## Slide 1
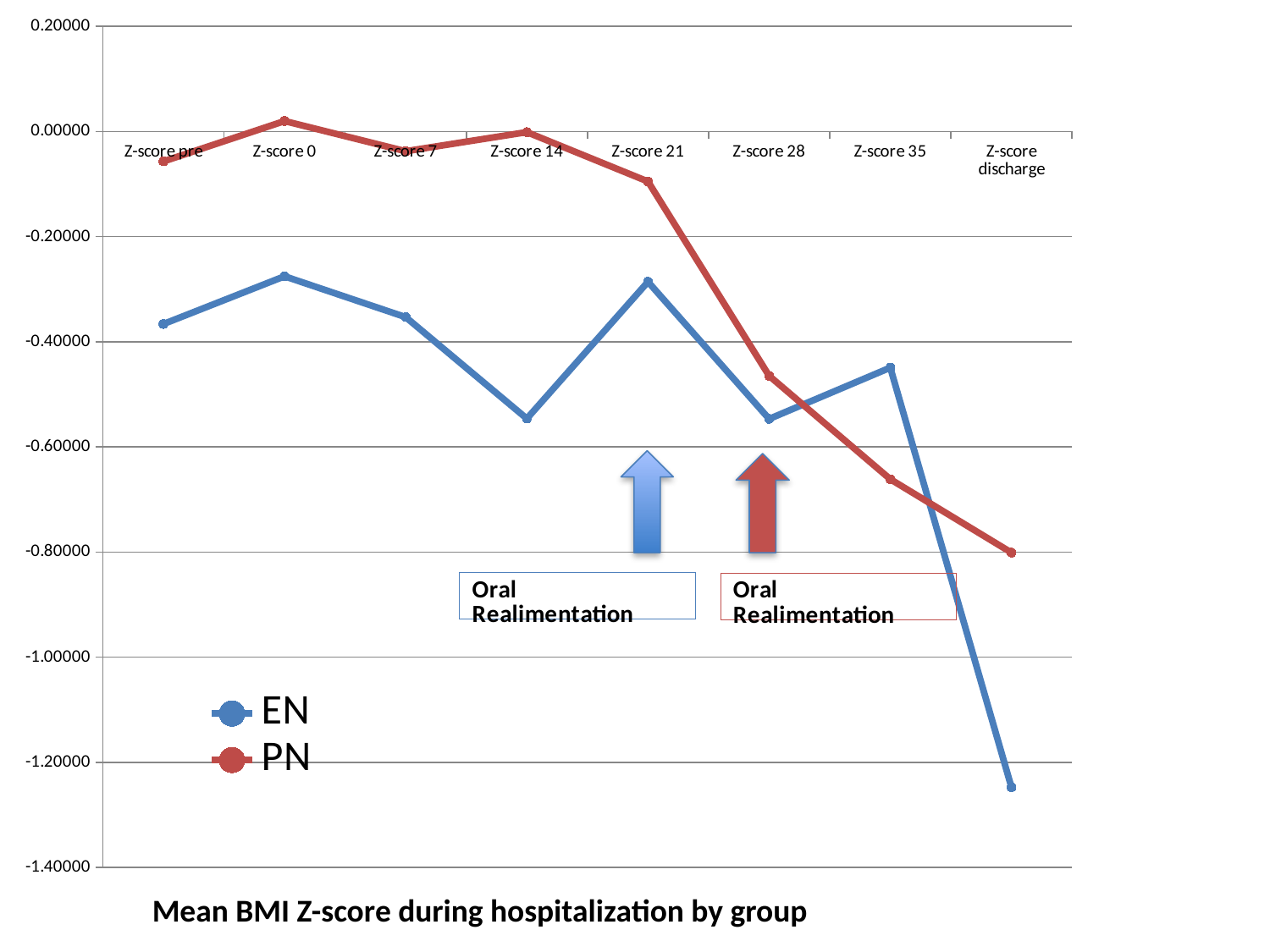

### Chart
| Category | EN | PN |
|---|---|---|
| Z-score pre | -0.3661539 | -0.0567857 |
| Z-score 0 | -0.2753846 | 0.02 |
| Z-score 7 | -0.3530769 | -0.0374074 |
| Z-score 14 | -0.5461538 | -0.0010714 |
| Z-score 21 | -0.2858333 | -0.095 |
| Z-score 28 | -0.5466667 | -0.4648 |
| Z-score 35 | -0.4491667 | -0.6615 |
| Z-score discharge | -1.2475 | -0.8011538 |Mean BMI Z-score during hospitalization by group
